# Exploring the Relationship Between Cyberchondria and Suicidal Ideation: Cross-Sectional Mediation Analysis

**DOI:** 10.2196/72414

**Published:** 2025-04-02

**Authors:** Richard Huan Xu, Xiao Liang, Vladan Starcevic

**Affiliations:** 1 Department of Rehabilitation Sciences Faculty of Health and Social Sciences Hong Kong Polytechnic University Hong Kong China (Hong Kong); 2 Faculty of Medicine and Health, Specialty of Psychiatry Nepean Clinical School, Sydney Medical School University of Sydney Sydney Australia; 3 Department of Psychiatry Nepean Hospital Penrith Australia

**Keywords:** cyberchondria, suicidal ideation, distress, structural equation modeling, mediation analysis

## Abstract

**Background:**

The proliferation of internet-based health information has intensified cyberchondria, or anxiety resulting from excessive health-related searches. The relationship between cyberchondria and suicidal ideation remains underexplored, although there are indications that people with high levels of cyberchondria may also be suicidal. Understanding this relationship is critical, given rising digital health-seeking behaviors and the need to mitigate suicide risk. Emerging evidence suggests that psychological distress can mediate the relationship between cyberchondria and suicidal ideation. However, to the best of our knowledge, no research has directly examined these associations.

**Objective:**

This study had two aims. The first was to examine the relationship between cyberchondria and suicidal ideation in a sample of the general Chinese population. The second aim was to investigate the possible role of psychological distress, reflecting the symptoms of depression and anxiety, as a mediator in the relationship between cyberchondria and suicidal ideation.

**Methods:**

Data were obtained from a cross-sectional and web-based survey conducted in 2024. Structural equation modeling analysis was used to assess the hypothesized association between cyberchondria and suicidal ideation, as well as the mediating effect of psychological distress on this association. The Cyberchondria Severity Scale-12 items, Suicidal Ideation Attributes Scale, and Kessler Psychological Distress Scale-10 items were used to measure cyberchondria, suicidal ideation, and psychological distress, respectively. Standardized (β) estimates, along with their 95% CIs, were calculated for all structural paths, adjusting for participants’ background characteristics.

**Results:**

A total of 2415 individuals completed the questionnaire (response rate=98.5%). Scores on the Cyberchondria Severity Scale-12 items ranged from 12 to 60, with the mean score being 40 (SD 7.9). The mean score on the Suicidal Ideation Attributes Scale was 12.7 (SD 9.9). Scores on the Kessler Psychological Distress Scale-10 items ranged from 10 to 50, and the mean score was 22 (SD 6.9). Cyberchondria, suicidal ideation, and psychological distress were significantly correlated. Structural equation modeling revealed a significant association between cyberchondria and psychological distress (β=.281; *P*<.001), between psychological distress and suicidal ideation (β=.504; *P*<.001), and between cyberchondria and suicidal ideation (β=.107; *P*<.001). The indirect effect of cyberchondria on suicidal ideation through psychological distress was also significant (β=.142; *P*<.001).

**Conclusions:**

The main contribution of this study is that it highlights an important relationship between cyberchondria and suicidal ideation, with a direct and statistically significant association between these variables. Their relationship is also mediated by psychological distress, which reflects the role of depressive and anxiety symptoms.

## Introduction

### Background

In today’s digital age, cybertechnology has had a mixed impact on suicidal tendencies [1]. On one hand, internet-based platforms can provide access to mental health resources, suicide prevention websites, crisis interventions, and peer support networks to help alleviate feelings of isolation and despair [1,2]. On the other hand, vulnerable individuals can be exposed to cyberbullying and harmful content, including prosuicide websites, which tend to increase the risk of suicidal ideation and behavior [1,3]. This reflects the complex interactions between digital environments and psychological processes, along with additional changes in the way that psychological distress and mental disorders manifest themselves.

### Cyberchondria and Suicidal Ideation

Cyberchondria is a good example of the transformative effect of cybertechnology on mental health. Cyberchondria refers to the anxiety-inducing practice of spending substantial time engaged in online health information seeking (OHIS) [4]. Cyberchondria is characterized by compulsive OHIS and high levels of health anxiety and, therefore, bears a resemblance to hypochondriasis [5,6]. Factors that drive repeated and excessive OHIS in cyberchondria include reassurance seeking and intolerance of uncertainty [7]. However, reassurance might never be experienced through OHIS, and uncertainty is not abolished, which only intensifies health anxiety and psychological distress. The experience can be overwhelming. For individuals caught in this cycle, the flood of conflicting internet-based information and the inability to find definitive answers can create a sense of helplessness, further amplifying their emotional and mental strain.

No study has yet investigated the relationship between cyberchondria and suicidal ideation. The potential link between them is rooted in a combination of psychological, behavioral, and emotional mechanisms that emerge from excessive and anxiety-driven OHIS. Central to this connection are self-reinforcing cycles of distress. Cyberchondria traps individuals in futile loops of searching, worrying, and repeated reassurance-seeking, which fail to resolve health concerns [8,9]. This cycle fosters helplessness and hopelessness, core components of suicidal thinking, as individuals perceive their situation as not improving, alongside a sense of entrapment—a recognized precursor to suicidal ideation [10]. The psychological toll of cyberchondria extends to cognitive overload [11], where the overwhelming volume of internet-based health information leads to mental exhaustion, impairing individuals’ ability to process what they find via the internet. The resultant frustration and despair are compounded by cyberchondria’s role in exacerbating chronic anxiety and depression [12], both well-established mediators of suicidal thoughts. Research indicates that anxiety and depression may amplify cyberchondria’s negative impact, as unresolved health worries and emotional dysregulation deepen feelings of inescapability [13,14]. Furthermore, individuals engaging in excessive OHIS may inadvertently stumble upon misinformation, exaggerated health risks, or even prosuicide material that normalizes or romanticizes ending one’s life [15]. These mechanisms create a pathway whereby cyberchondria heightens vulnerability to suicidal ideation.

Further support for the link between cyberchondria and suicidal ideation comes from some theories of suicidality. For example, the Cry of Pain model attributes suicidality to defeat, entrapment, and lack of rescue [16]. In the context of cyberchondria, defeat may be experienced as ongoing and relentless health anxiety despite efforts to mitigate it, entrapment may refer to unavoidable and compulsive cycles of OHIS, and lack of rescue may be experienced through the perception that there is no support to show a way out. Likewise, the three-step theory posits that pain, hopelessness, and disconnection are causally related to suicidal ideation [17]. With regard to cyberchondria, ineffective or frustrating OHIS may contribute to emotional pain and hopelessness, while disconnection may arise from social isolation stemming from excessive time devoted to OHIS at the expense of in-person interactions.

### Objectives

This study aims to examine the relationship between cyberchondria and suicidal ideation in a sample of the general Chinese population through structural equation modeling (SEM) analysis. We also examined a possible role for psychological distress, reflecting the symptoms of depression and anxiety, as a mediator in the relationship between cyberchondria and suicidal ideation. On the basis of the aforementioned research findings and theoretical considerations and observations [18], we hypothesized that cyberchondria and suicidal ideation will be directly related and that psychological distress will mediate this relationship.

## Methods

### Study Design, Participants, and Data Collection

Data were collected using a cross-sectional and web-based survey that was conducted between April 2024 and May 2024. Participants were recruited from Wenjuanxing, a Chinese survey platform with an internet-based panel of 2.6 million members. The company targets users by demographic, geographic, or behavioral criteria, matching survey needs and engaging panelists via email, in-app alerts, and reward campaigns (eg, cash and vouchers). Quality is ensured through dynamic filtering, fraud detection, and panel segmentation to optimize representative sampling. Individuals who were 18 years or older, permanent residents of China, could read Chinese, and were able to provide informed consent were eligible to participate in the survey. All eligible panelists were invited to complete a series of questionnaires. The first section of the survey was about informed consent. The participants were required to read this section and agree to it if they wanted to proceed with the survey. The panelists who provided their consent were then asked to complete four questionnaires that collected information on their demographics, socioeconomic status, cyberchondria, psychological distress, and suicidal ideation. All instruments used in this study were validated for internet-based administration [19-21]. The research team collaborated with the survey company to implement a series of techniques to ensure the quality of the data. This included using CAPTCHA (Completely Automated Public Turing Test to Tell Computers and Humans Apart) to prevent bots from submitting responses, conducting time analysis, excluding responses submitted quicker than a reasonable time (less than 5 minutes), allowing only one submission from a single IP address within a given time frame, and identifying and filtering out any response patterns that appeared to be generated in parallel.

### Measures

#### Cyberchondria

The severity of cyberchondria was measured using the Cyberchondria Severity Scale-12 items (CSS-12). The CSS-12 has been found to have psychometric properties as good as the original version and has been validated among Chinese populations [22]. The items on the CSS-12 are scored on a Likert-type scale ranging from 1 (“never”) to 5 (“always”). The total score ranges from 12 to 60, and higher scores indicate higher levels of cyberchondria. The internal consistency of the CSS-12 in this study was very good (Cronbach α=0.85).

#### Suicidal Ideation

Suicidal ideation was assessed using the Suicidal Ideation Attributes Scale (SIDAS). The SIDAS is an instrument that screens individuals for suicidal thoughts and assesses the severity of those thoughts [23]. It comprises five items, each focusing on a specific aspect of suicidal thoughts: frequency, controllability, closeness to attempt, level of distress associated with the thoughts, and impact on daily functioning. The items are rated on an 11-point scale, from 0 (“never”) to 10 (“always”). Total scores range between 0 and 50, with higher scores indicating more severe suicidal thoughts. The SIDAS is valid and reliable for use among the Chinese population [20]. The internal consistency of the SIDAS in this study was good (Cronbach α= 0.83).

#### Psychological Distress

Psychological distress was measured with the Kessler Psychological Distress Scale-10 items (K10). This tool is used to screen for psychological distress, including anxiety and depressive symptoms [24]. It comprises 10 items assessing symptoms from the past month or the worst month of the past year. The items are rated on a 5-point Likert scale ranging from 1 (“none of the time”) to 5 (“all of the time”). The total score ranges from 10 (indicating no distress) to 50 (indicating severe distress). The K10 has been found to have good psychometric properties among the Chinese population [25]. The internal consistency of the K10 in this study was very good (Cronbach α=0.88).

### Data Analysis

Descriptive statistics were used to describe participants’ demographic characteristics. Continuous variables (eg, age) were denoted using means and SDs. Categorical variables (eg, sex) were denoted using frequencies and percentages. Pearson correlation coefficients (*r*) were used to determine associations between variables, with *r*≥0.3 and *r*≥0.5 indicating moderate and large effects, respectively [26]. We used a backward elimination approach with the linear regression model to examine the direct association between cyberchondria and suicidal ideation, and standardized coefficients (β) and 95% CIs were calculated.

The hypothesized model was assessed using SEM analysis with the maximum likelihood estimation method. Cyberchondria was identified as the observed variable (exposure) and suicidal ideation as the outcome variable. The magnitude of the mediating effect (of psychological distress) was also estimated. Standardized (β) and unstandardized (B) estimates, along with their 95% CIs, were calculated for all structural paths after adjusting for demographic characteristics (potential confounders), including sex, age, education level, marital status, employment status, and family registry (urban or rural dwelling). Errors for mediator and outcome variables were added to the model as covariates. Model performance was assessed using the comparative fit index (>0.95), Tucker-Lewis Index (>0.95), root mean square error of approximation (<0.05), and standardized root mean squared residual (<0.08) [27]. All statistical analyses were performed using R software (R Core Team), and a 2-sided *P* value below .05 denoted statistical significance.

### Ethical Considerations

The study protocol and informed consent process were approved by the Institutional Review Board of Hong Kong Polytechnic University (Ref ID: HSEARS20230502006) on May 15, 2023. Informed consent was obtained from all participants. All data were deidentified, and no compensation was provided to participants by the research team.

## Results

### Participants’ Characteristics

Table 1 presents the demographic characteristics of the participants. A total of 2415 individuals (response rate=98.5%) completed the questionnaires and provided valid responses. Among them, 51.4% (1242/2415) were female, 76% (1836/2415) were married, and 82.5% (1992/2415) had completed tertiary education. Respondents’ ages ranged from 18 to 80 years, with the average age being 32.9 years. In addition, approximately 77.1% (1861/2415) of respondents reported a perceived socioeconomic status equal to the local average.

**Table 1:** Respondents’ demographic characteristics (N=2415).

Characteristics		Values
**Sex, n (%)**		
	Male	1173 (48.6)
	Female	1242 (51.4)
**Educational level, n (%)**		
	Secondary or below	423 (17.5)
	Tertiary	1992 (82.5)
**Family registry, n (%)**		
	Urban	1200 (49.7)
	Rural	1215 (50.3)
**Marital status, n (%)**		
	Married	1836 (76)
	Nonmarried	579 (24)
**Employment, n (%)**		
	Active	2182 (90.4)
	Nonactive	233 (9.6)
**Perceived socioeconomic status, n (%)**		
	Lower than local average	225 (9.3)
	Equal to local average	1861 (77.1)
	Higher than local average	329 (13.6)
Age (years), mean (SD; range)		32.9 (7.7; 18-80)

### Scores on the CSS-12, SIDAS, and K10, and Correlations Among Variables

In this study, scores on the CSS-12 ranged from 12 to 60, with the mean score being 40 (SD 7.9; Table 2). The mean score on the SIDAS was 12.7 (SD 9.9). Scores on the K10 ranged from 10 to 50, and the mean score was 22 (SD 6.9). Furthermore, cyberchondria, suicidal ideation, and psychological distress were significantly correlated. The Pearson correlation coefficient between psychological distress and suicidal ideation was the highest at *r*=0.46 (*P*<.001). In addition, higher levels of cyberchondria were found to be associated with increased levels of distress and suicidal ideation (*r*=0.23 and *r*=0.21, respectively, both *P*<.001).

**Table 2:** The profiles of measures and the correlations between measures.

Instrument	Score				Cronbach α		Pearson correlation coefficient		
	Mean (SD)	Median (IQR)	Range			CSS-12^a^		SIDAS^b^	K10^c^
CSS-12	40 (7.9)	41 (11)	12-60	0.85		1		0.21^d^	0.23^d^
SIDAS	12.7 (9.9)	8 (11)	5-54	0.83		0.21^d^		1	0.46^d^
K10	22.0 (6.9)	21 (9)	10-50	0.88		0.23^d^		0.46^d^	1

^a^CSS-12: Cyberchondria Severity Scale-12 items.

^b^SIDAS: Suicidal Ideation Attributes Scale.

^c^K10: Kessler Psychological Distress Scale-10 items.

^d^*P*<.001

### Direct Association Between Cyberchondria and Suicidal Ideation

Table 3 displays the results of the multivariate linear regression analysis. The models revealed a statistically significant association between cyberchondria and suicidal ideation (β=.166, 95% CI 0.135-0.197), even after adjusting for participants’ demographic variables (β=.165, 95% CI 0.134-0.196). Older age was associated with a lower level of cyberchondria, while a higher education level was associated with a higher level of cyberchondria.

**Table 3:** The relationship between cyberchondria and suicidal ideation.

Characteristics		β (95% CI)	
		Model 1^a^	Model 2^b^
Suicidal ideation		0.166 (0.13 to 0.19)	0.165 (0.13 to 0.19)
Age		—^c^	–0.07 (–0.12 to –0.03)
**Education level**			
	Secondary or below	—	Reference
	Tertiary	—	1.41 (0.58 to 2.25)
**Marital status**			
	Married	—	Reference
	Nonmarried	—	1.02 (0.20 to 1.85)

^a^Model 1: univariable model that only suicidal ideation is included as an independent variable.

^b^Model 2: multivariable model that suicidal ideation and all the demographic variables are included in the model using stepwise method.

^c^Not applicable.

### Model Performance and Factor Loadings

The hypothesized mediation model demonstrated an acceptable fit (Table 4), with comparative fit index=0.913, Tucker-Lewis Index=0.904, root mean square error of approximation=0.046, and standardized root mean squared residual=0.045. All items loaded significantly onto the latent variables (Table 5). The β ranged from 0.578 to 0.716 (*P*<.001) for psychological distress, from 0.471 to 0.652 (*P*<.001) for cyberchondria, and from 0.173 to 0.904 (*P*<.001) for suicidal ideation.

**Table 4:** Model fit indicator.

Model fit	Value
CFI^a^	0.909
TLI^b^	0.903
RMSEA^c^	0.047
SRMR^d^	0.052

^a^CFI: comparative fit index.

^b^TLI: Tucker-Lewis Index.

^c^RMSEA: root mean square error of approximation.

^d^SRMR: standardized root mean squared residual.

**Table 5:** Factor loadings for measures and model performance.

Measure and items			Β (95% CI)		SE
**K10^a^**					
	K10-1	0.577 (0.548-0.606)		0.015	
	K10-2	0.599 (0.57-0.627)		0.014	
	K10-3	0.692 (0.668-0.716)		0.012	
	K10-4	0.717 (0.695-0.74)		0.011	
	K10-5	0.585 (0.556-0.614)		0.015	
	K10-6	0.684 (0.66-0.708)		0.012	
	K10-7	0.655 (0.629-0.68)		0.013	
	K10-8	0.708 (0.686-0.731)		0.012	
	K10-9	0.624 (0.597-0.651)		0.014	
	K10-10	0.685 (0.661-0.709)		0.012	
**CSS-12^b^**					
	CSS1	0.553 (0.522-0.584)		0.016	
	CSS2	0.579 (0.549-0.609)		0.015	
	CSS3	0.546 (0.514-0.578)		0.016	
	CSS4	0.652 (0.626-0.679)		0.014	
	CSS5	0.464 (0.429-0.498)		0.018	
	CSS6	0.595 (0.565-0.624)		0.015	
	CSS7	0.593 (0.564-0.623)		0.015	
	CSS8	0.616 (0.588-0.645)		0.015	
	CSS9	0.613 (0.584-0.642)		0.015	
	CSS10	0.553 (0.522-0.584)		0.016	
	CSS11	0.47 (0.436-0.505)		0.018	
	CSS12	0.488 (0.454-0.522)		0.017	
**SIDAS^c^**					
	SIDAS1	0.904 (0.895-0.912)		0.005	
	SIDAS2	0.173 (0.133-0.213)		0.02	
	SIDAS3	0.871 (0.86-0.882)		0.006	
	SIDAS4	0.934 (0.927-0.941)		0.004	
	SIDAS5	0.891 (0.881-0.901)		0.005	
**CSS-12**					
	Sex	–0.035 (–0.078 to 0.008)		0.022	
	Age	–0.107 (–0.158 to –0.057)		0.026	
	Family registry	–0.034 (–0.079 to 0.011)		0.023	
	Employment	–0.012 (–0.062 to 0.038)		0.026	
	Marital status	0.053 (0-0.106)		0.027	
	Education	0.057 (0.012-0.102)		0.023	

^a^K10: Kessler Psychological Distress Scale-10 items.

^b^CSS-12: Cyberchondria Severity Scale-12 items.

^c^SIDAS: Suicidal Ideation Attributes Scale.

### Results of the Hypothesized Mediation Model

**Figure 1:**
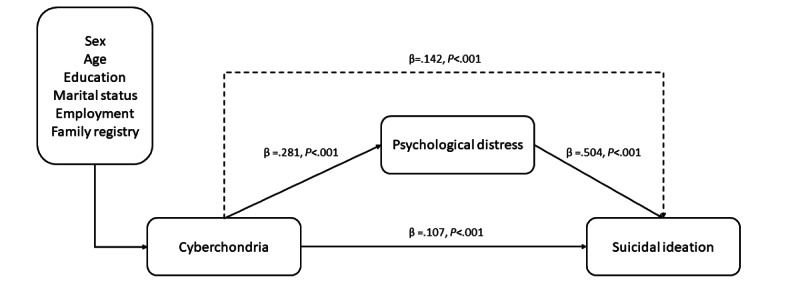
The structural equation modeling of the association between cyberchondria and suicidal ideation mediated via psychological distress, with adjustment for sex, age, education level, marital status, employment status, and family registry. The standardized coefficients of structural paths are shown and all paths were statistically significant. Error covariances are not presented for clarity.

The SEM model is shown in Figure 1. After adjusting for sex, age, education level, marital status, employment status, and family registry, all direct effects were found to be statistically significant. There was a significant direct relationship between cyberchondria and psychological distress (β=.281; *P*<.001), between psychological distress and suicidal ideation (β=.504; *P*<.001), and between cyberchondria and suicidal ideation (β=.107; *P*<.001). The indirect effect of cyberchondria on suicidal ideation through psychological distress was also significant (β=.142; *P*<.001). This suggests that psychological distress increases the impact of cyberchondria on suicidal ideation.

## Discussion

### Principal Findings

The rapid increase in the use of the internet, coupled with the widespread availability of internet-based health information, has made cyberchondria a relevant topic for internet users worldwide. Although studies have demonstrated that cyberchondria has negative effects on mental health [28,29], to the best of our knowledge, this is the first study to explore the direct relationship between cyberchondria and suicidal ideation. With a large sample, this study provides empirical evidence of the association between high levels of cyberchondria and a high risk of suicidal ideation. This finding is novel and crucial, with important clinical implications. However, the key issue is understanding the mechanisms that may account for the relationship between cyberchondria and suicidal ideation, particularly whether these mechanisms involve other psychopathology as opposed to involving cyberchondria itself.

One mechanism may implicate health anxiety, which typically accompanies cyberchondria. Studies in individuals with hypochondriasis, a condition characterized by high levels of health anxiety, are also relevant here because of the close link between cyberchondria and hypochondriasis [4]. Mataix-Cols et al [30] found that individuals with hypochondriasis faced an increased risk of unnatural death, particularly suicide, compared with those without hypochondriasis. Similarly, Noyes et al [31] reported a higher prevalence of lifetime suicide attempts in individuals with hypochondriasis than in those without hypochondriasis. Therefore, the relationship between cyberchondria and suicidal ideation might be explained, at least to some extent, by the high levels of health anxiety that usually characterize cyberchondria.

This study found a direct and statistically significant association between cyberchondria and suicidal ideation, prompting a question as to whether there is something about cyberchondria itself that might explain this association. Although we could not investigate this question directly, it is possible that repetitive, frustrating, and at times exhausting internet-based health searches lead to suicidal ideation. Finding frightening internet-based health content might have the same effect by inducing a sense of hopelessness, given that hopelessness is a well-established risk factor for suicide [32]. Furthermore, intolerance of uncertainty, which often drives OHIS [7], may also act as a risk factor for increased suicidal ideation because intolerance of uncertainty has been linked with suicide [33].

Some studies have indicated a significant association between cyberchondria and internet-based misinformation, which can significantly increase suicide risk. For instance, misinformation about mental health treatments can lead individuals to pursue ineffective or harmful interventions, which can exacerbate their condition and heighten their feelings of hopelessness and despair [34]. Misinformation can also perpetuate mental health stigma, making individuals less likely to seek help or openly discuss their struggles [35]. Yet another explanation for the association between cyberchondria and suicidal ideation may involve problematic internet use, which has been demonstrated to have an important relationship with cyberchondria [36]. Problematic internet use encompasses internet-based behaviors that may increase the risk of self-harm and suicide, such as cyberbullying [37] and problematic use of social networking sites [38].

Our mediation analysis revealed that depression and anxiety, represented by the construct of psychological distress, mediated the relationship between cyberchondria and suicidal ideation. This is not a surprising finding, considering that depression and anxiety are well-known risk factors for suicidal ideation and suicide [39]. The temporal relationships between cyberchondria, depression, and anxiety may be complex, but their interplay is likely to cause or exacerbate suicidal ideation.

### Implications for Public Health, Clinical Practice, and Further Research

Our results confirm that cyberchondria represents a significant public health problem and poses further challenges to mental health. In view of the relationship between cyberchondria and suicidal ideation, as well as the role played by other psychopathology in this relationship, we recommend early screening for cyberchondria and cooccurring mental disorders to facilitate their identification and treatment. Health care providers should regularly follow up with individuals who have cyberchondria and reassess their suicide risk over time. Our findings also support public health campaigns to raise awareness of cyberchondria and its negative consequences. Finally, clinical practice should incorporate the notion that prevention and management of cyberchondria may also contribute to suicide prevention efforts.

We present the following directions for future research. First, any causal relationship between cyberchondria and suicidal ideation should be explored. Although cyberchondria may be more likely to heighten suicidal ideation, the latter may also contribute to cyberchondria. Prospective studies and longitudinal analyses are necessary to clarify these relationships. Second, this study found a significant mediating effect of psychological distress. Given that psychological distress is a broad construct that refers to a state of emotional suffering and is associated with various mental disorders, it is crucial to carefully assess all relevant, cooccurring psychopathology, not only depression and anxiety. This would allow for a better understanding of the interplay between cyberchondria and these domains of psychopathology, and how that interplay relates to suicidal ideation. Third, future research should focus on the direct links between cyberchondria and suicidal ideation, controlling for the effects of other conditions. Finally, research should be conducted on individuals with cyberchondria across different ages and education levels, as these variables may affect the relationship between cyberchondria and suicidal ideation.

### Limitations

Several limitations should be addressed. First, the cross-sectional design restricted the exploration of temporal changes in cyberchondria levels and suicidal ideation. This made it challenging to establish causal relationships among the variables. Despite this, our findings establish a critical foundation for future longitudinal studies to investigate directionality and underlying mechanisms. Second, mostly younger and highly educated individuals participated in the survey. This could introduce a selection bias and complicate the generalization of our findings. However, this demographic represents a key population for studying internet-based health resource use, as they are major users of such platforms. While broader sampling is needed, this study establishes an initial framework for investigating this relationship in more diverse cohorts. Finally, the K10 was used to assess psychological distress, which is not a specific construct nor a clinical diagnosis. Also, several relevant variables, such as levels of depression and anxiety and intensity of problematic internet use, were not assessed directly in the present study. However, although our use of the K10 as a proxy for assessing depressive and anxiety symptoms was not ideal, we had to ensure that the number of instruments administered via the internet did not have a major impact on the willingness to participate in the survey. Despite these trade-offs, the study advances theoretical understanding and offers practical insights, such as leveraging brief distress screenings to identify at-risk individuals, while indicating the need for future work with specific instruments.

### Conclusions

The study’s key contribution lies in its identification of a direct, statistically significant association between cyberchondria and suicidal ideation, mediated by psychological distress (eg, depressive and anxiety symptoms). Its key limitations include a cross-sectional design, the use of a broad measure of psychological distress, and skewed demographic characteristics of study participants. Despite these limitations, this study reveals a hitherto unknown relationship and points to future research that should have a longitudinal design, be conducted in diverse samples, and use more specific instruments. The findings of the study hold immediate relevance by highlighting cyberchondria’s detrimental effects and advocating for its prevention, early detection, and management. Finally, by raising awareness of cyberchondria’s link to suicidality, this research provides a critical foundation for addressing an urgent public health issue.

## Abbreviations

CAPTCHA: Completely Automated Public Turing Test to Tell Computers and Humans Apart
CSS-12: Cyberchondria Severity Scale-12 items
K10: Kessler Psychological Distress Scale-10 items
OHIS: online health information seeking
SEM: structural equation modeling
SIDAS: Suicidal Ideation Attributes Scale

## Data Availability

The datasets generated or analyzed during this study are not publicly available but are available from the corresponding author on reasonable request.
